# Evaluating Effectiveness of Multi-Component Waste Plastic Bags on Bitumen Properties: Physical, Rheological, and Aging

**DOI:** 10.3390/polym16121669

**Published:** 2024-06-12

**Authors:** Nioushasadat Haji Seyed Javadi, Soheil Heydari, Ailar Hajimohammadi

**Affiliations:** School of Civil and Environmental Engineering, University of New South Wales, Sydney, NSW 2052, Australia

**Keywords:** asphalt binder, modified bitumen, waste plastic bags, cross-contamination of plastics, polyethylene

## Abstract

This study examines the applicability of an unknown composition waste plastic bag sample as bitumen modifier. The waste components were initially characterized to identify the type of plastics and the level of impurity. Asphalt binder performance was examined for rutting, thermal, and age resistance. The results revealed that the waste plastic bags, predominantly consisted of Low-Density Polyethylene (LDPE) and Linear Low-Density Polyethylene (LLDPE) and contained 6.1% impurities. The binder tests indicated that the waste plastic bags enhanced the rutting resistance of bitumen by one grade, with its modification more similar to LLDPE, rather than LDPE. The thermal degradation and aging properties of the modified binders demonstrated that the bitumen modified by the waste plastic bags exhibited slightly lower resistance to temperature and aging compared to virgin LDPE and LLDPE. This was attributed to the impurities contained in the waste plastic. In conclusion, the analyzed waste plastic bags proved to be suitable for use in binder modification, presenting a viable alternative to virgin LLDPE.

## 1. Introduction

The continuous production of plastics over several decades has led to the generation of substantial plastic waste worldwide [1,2]. A global material balance study on plastics highlights that 79% of the total plastics produced worldwide end up in the environment as waste, with only 9% being recycled [3]. Many plastic materials, such as plastic bags, are used for a short time and, being non-biodegradable, have a significant environmental impact [4]. They eventually become waste, ending up in oceans and human food chains [5]. The growing global concern regarding plastic waste and environmental sustainability has prompted innovation in the field of construction materials [6,7,8]. In response to this scenario, extensive research has been conducted on reusing waste plastics to produce construction materials, reducing the volume of plastic waste and alleviating the increasing demand for natural resources in the construction industry [9,10]. Among the numerous applications of waste plastics in construction, their utilization in modifying asphalt binders has gained significant attention [11,12,13].

The literature highlights the advantageous role of waste plastics in enhancing the performance of asphalt binder [14,15,16,17]. The plastic products are categorized into seven groups as High-Density Polyethylene (HDPE), Low-Density Polyethylene (LDPE), Polypropylene (PP), Polyethylene Terephthalate (PET), Vinyl/Polyvinyl Chloride (PVC), Polystyrene (PS), and OTHER (other kinds of plastics) [18]. Among them, polyethylene and polypropylene account for the majority of the waste plastic streams. Numerous previous studies have investigated the suitability of single plastic types within these groups, demonstrating their significant impact on enhancing the properties and performance of bitumen, particularly in terms of rutting and aging [19,20,21,22,23]. Nevertheless, real-world plastic products are not made of a single plastic polymer. To meet the needs of end-user applications, creating multilayers of plastics through processes such as lamination or coextrusion are required [24,25]. Moreover, various organic and inorganic additives may be integrated into the polymer matrix, depending on the intended application, such as optimizing the properties of the final product or reducing production costs [26]. Considering plastic bags, for instance, which represent one of the most used types of plastic materials, with a consumption of around 500 billion plastic bags yearly worldwide [27], they typically consist of a blend of multiple plastics and include organic and inorganic additives [28]. Upon disposal, recycling processes encounter difficulties in fully segregating and recycling the diverse components of these products [29,30,31], resulting in their accumulation in landfills [4,32]. Utilizing these products for bitumen modification introduces the potential for each component to impact the properties and performance of the bitumen differently, contributing to variations in the final performance of asphalt mixtures.

In this study, a sample of waste plastic bags, supplied by State Asphalt New South Wales (the industry partner of the project (https://www.stateasphalts.com.au [33] (accessed on 16 January 2024)), was selected for compositional analysis. The objective was to evaluate the potential variations that the waste might introduce in bitumen performance once employed as a bitumen modifier, considering its diverse composition. Comprehensive characterization tests were conducted on the waste sample to determine its actual composition. Subsequently, bitumen modification was performed using both the waste plastic sample and the virgin form of the polymers found in the waste plastic. The physical, rheological resistance to rutting and aging and thermal stability of the resultant modified bitumen sample were investigated. This approach aimed to identify any variations between the modification by the waste sample and its virgin plastic counterparts.

## 2. Materials and Methods

### 2.1. Experimental Plan and Statistical Data Processing

In this study, 3 kg of shredded post-consumer waste plastic bags with unknown composition were provided by the industry partner. To obtain representative samples of the waste, it was thoroughly mixed by hand and then sub-divided into four smaller portions, randomly selecting samples from these portions for analysis. Each sub-sample was characterized using Differential Scanning Calorimetry (DSC), Attenuated Total Reflectance Fourier Transform Infrared Spectroscopy (FTIR), and Thermogravimetric Analysis (TGA) to determine its composition, identifying the plastic types and impurities present. The average of the results was reported.

Following this, samples of modified bitumen using the four sub-samples were prepared. All samples were tested for rutting and aging resistance, and the results were averaged from the modifications with each sub-sample. To evaluate the effectiveness of the waste plastic, samples of modified bitumen using the virgin form of the plastics found in the composition were also produced for comparison. The asphalt binder tests used to examine rutting performance included conventional tests (softening point, penetration, viscosity), a Dynamic Shear Rheometer (DSR), and the Multiple Stress Creep Recovery Test (MSCR). For aging properties, samples were aged using a Rolling Thin Film Oven (RTFO) and tested using DSR to plot black diagrams, allowing for the comparison of the crossover modulus before and after aging to measure the effect of aging on the binder properties.

### 2.2. Materials

**Figure 1:**
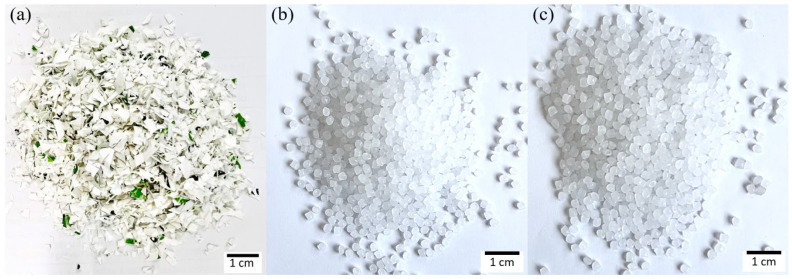
Camera image of shredded waste plastic bags (**a**), virgin LDPE pellets (**b**), virgin LLDPE pellets (**c**).

Bitumen Class C170, a standard asphalt binder in Australia, was chosen as the base bitumen and sourced from Viva Energy Pty Ltd, Sydney, Australia. This bitumen type is equivalent to the globally used bitumen penetration grade 70/100. The viscosity of the Class C170 bitumen at 60 °C is approximately 170 Pa·s, according to the Australian bitumen specification [34]. The waste plastic bags sample was received in shredded form (Figure 1) from the industry partner of the project, State Asphalts NSW (https://www.stateasphalts.com.au (accessed on 16 January 2024)). The common plastic used for manufacturing plastic bags is polyolefin, which is comprised of High-Density Polyethylene (HDPE), Low-Density Polyethylene (LDPE), Linear Low-Density Polyethylene (LLDPE), and Polypropylene (PP). Therefore, the virgin form of the four polymers was used in this study as a reference for characterizing the composition of the waste plastic bags. The virgin plastics were supplied by Primaplas Pty Ltd, Sydney, Australia. in pellets, and their reported properties are detailed in Table 1.

### 2.3. Characterization of the Waste Plastic Bags

#### 2.3.1. Thermal Behavior Analysis

DSC was used to analyze the composition of the waste sample via a DSC instrument (NETZSCH DSC 204F1Phoenix, Garbsen, Germany). DSC is a technique used to investigate the polymer’s response to heating, which distinguishes between various components based on their different melting temperatures [35].

The DSC test was conducted on the waste sample and the virgin plastics, utilizing aluminum crucibles with pierced lids. The test comprised of two cycles of heating and cooling. The first cycle was conducted to eliminate any thermal history effects. The second heating cycle was used to measure the melting temperature of the samples [36]. The temperature range was set from 25 to 200 °C. Initially, the samples were equilibrated at 25 °C for 5 min. Subsequently, the temperature was increased at a heating rate of 10 °C/min until reaching 200 °C, where it was maintained for 3 min. Subsequently, the samples were cooled down to 25 °C at a cooling rate of 10 °C/min. The temperature was again held at 25 °C for 3 min, marking the completion of the first cycle [37].

DSC was also used to measure the degree of crystallization of the virgin and the waste plastics. This involves drawing a linear baseline from the first onset of the melting to the last trace of crystallinity. The melting enthalpy of the sample is calculated from the area under this endotherm. The crystallinity level is then defined using Equation (1) [38]:

(1) Xc=∆Hm∆Hm0×100%

where $∆H_{m}$ is the melting enthalpy of the sample and ${∆H}_{m0}$ is the melting enthalpy of 100% crystalline polymer. The ${∆H}_{m0}$ for polyethylene was taken as 293 J/g [39].

#### 2.3.2. Chemical Characterization Analysis

ATR-FTIR was used as a complementary technique to identify the compositions of the waste samples, using a FTIR instrument (Perkin-Elmer FTIR Spectrum Two, Beaconsfield, Buckinghamshire). The spectrometer is equipped with a diamond crystal ATR cell. The ATR crystal was cleaned with a 70% 2-propanol solution before and after testing each sample. Before scanning a sample, the background spectrum was scanned [40]. To ensure a proper contact between the sample and crystal, it was compressed against the sample with a force of at least 80 N. For each sample, 32 scans were collected with a resolution of 4 cm^−1^ over the region of 4500–450 cm^−1^ wavenumber against air as the background [41].

#### 2.3.3. Thermal Stability Analysis

TGA was utilized to assess the presence of both organic and inorganic additives in the waste sample and determine its purity compared to virgin plastics. TGA was also employed to study the thermal degradation behavior of asphalt binders and to examine how the modifications have influenced thermal stability. The TGA analysis was carried out using a TG/DSC instrument (TA Instruments SDT Q600, New Castle, DE, USA) employing an alumina crucible. Each analysis consisted of exposing samples weighing between 10 to 20 mg to a temperature range from room temperature to 800 °C. This temperature increase occurred at a consistent rate of 10 °C/min, within a nitrogen atmosphere with a flow rate of 50 mL/min to prevent oxidation [42]. A reference point was established using an empty crucible during the analysis. The decomposition of the samples was monitored and measured concerning temperature, providing valuable insights into their thermal characteristics and composition. The corresponding derivative thermogravimetric (DTG) curve was also studied to record the temperature at which the sample’s weight loss is at the maximum rate (T_max_).

### 2.4. Preparation of Polymer Modified Bitumen Samples

This study explored the impact on bitumen modification of the shredded waste plastic bags sample and its virgin components using LDPE and LLDPE pellets. As the properties and performance of the final polymer-modified bitumen are significantly influenced by mixing conditions, temperature, duration, and mixing speed [43,44], the mixing method was initially optimized and then kept constant for preparing all modified binders. Following a series of trials testing various mixing conditions, it was established that the optimal conditions, ensuring sufficient flow in the mixture, were a speed of 3000 rpm, a mixing time of 120 min, and a mixing temperature of 160 °C. Employing these conditions, modified binders were produced. The concentration of the modifier was 5% per weight of bitumen for all samples. The compositions of the samples and their names are listed in Table 2.

### 2.5. Aging Procedure of the Samples

All samples were subjected to aging in a RTFO (CONTROLS, Milan, Italy). This procedure simulates the short-term aging of binders occurring during the production and placement of asphalt mixtures [45,46].Short-term aging is characterized by oxidation and volatilization occurring at elevated temperatures in the presence of hot air during mix production [47]. The aging process was performed according to AASHTO T240 [48], which requires heating at 163 °C for 85 min. The aged samples were then analyzed to assess the impacts of short-term aging on the rheological properties. The crossover modulus before and after aging was measured to characterize the aging effect. This provided a comprehensive understanding of the effectiveness of waste plastic compared to its virgin counterparts.

### 2.6. Conventional Properties of Asphalt Binders

To measure the consistency of asphalt binders, a penetration test was performed using a penetrometer (CONTROLS Penetrometer, Milan, Italy). Higher penetration values indicate a softer binder [49]. The test procedure was conducted according to Australian standard AS 2341.12 [50]. To determine the penetration value, a needle with a 100 g load was applied vertically into the bitumen for five seconds at 25 °C. The penetration value was calculated based on the amount of needle movement in tenths of a millimeter (0.1 mm). For enhanced accuracy, for each asphalt binder, three samples were measured, and the average of three penetration values was reported.

To determine the softening temperature of asphalt binders, the softening point test was conducted using ring and ball apparatus. The softening point value indicates the temperature at which the bitumen achieves a specific level of softening [51,52]. The softening point provides information about the susceptibility of the asphalt binder to high temperatures. Higher softening point values indicate greater resistance to hot conditions [53]. The test was conducted according to the Australian standard AS 2341.18 [54]. Two samples of each asphalt binder were tested, and the average of the two was reported. In cases where the difference between the values exceeded 1 °C, the test was repeated [54].

To assess the resistance of the samples to flow, a viscosity test was conducted using a Brookfield viscometer (AMETEK Brookfield LVDV2T viscometer, Berwyn, PA, USA). The test was in accordance with AS 2341.4 [55]. The viscosity test is vital for assessing polymer-modified bitumen, indicating its resistance to shearing deformation. The test was carried out at 135 °C. As per the standard protocol, 10.5 g of binder was poured into the viscometer’s tube, and a spindle (specifically, #27 in this study) was introduced into the tube and rotated. The viscometer measured the resistance exhibited by the bitumen samples. Viscosity values were recorded at 1 min intervals until a consistent reading was achieved. The average of the last two readings was reported.

### 2.7. Rheological Analysis of Asphalt Binders

Bitumen is a viscoelastic material that exhibits time and temperature dependent responses to applied stresses [56]. In terms of rheology, bitumen behaves like an elastic solid at rapid loading (high loading frequency–short loading time) and/or low temperatures and acts like a viscous fluid when subjected to slow loading (low frequency–long loading time) and/or under high temperatures [57]. To examine the rheological behavior of the asphalt binders, temperature and frequency sweep tests using a DSR instrument (NETZSCH Kinexus DSR, Selb, Germany) were performed. By measuring the viscoelastic parameters of asphalt binders at various temperatures and frequency ranges, including loss modulus (G”), storage modulus (G’), complex modulus (G*), and phase angle (δ), DSR provides valuable insights about the rutting resistance and flow properties of asphalt binders [58].

#### 2.7.1. High-Temperature Rutting Resistance of Asphalt Binders

1. Superpave rutting parameter

The Superpave rutting parameter (G*/sinδ) is a factor that represents the stiffness of asphalt binders at high temperatures [59]. A higher rutting parameter is an indication of the greater performance of the binder against permanent deformation and rutting. To measure the rutting parameter, temperature sweep tests from 64 to 76 °C with an increment of 6 °C were conducted according to the AASHTO T315 test method [60]. Frequency was kept constant at 10 rad/s. The software of the DSR machine recorded the complex shear modulus (G*) and phase angle (δ) to calculate the rutting resistance parameter (G*/sinδ). Based on the performance grading system, an asphalt binder is appropriate for a specific high temperature grade when the rutting parameter of the unaged binder is higher than 1 kPa and greater than 2.2 kPa for the aged binder at that temperature. The DSR increased the temperature until the sample met the failure criteria (unaged: G*/sinδ ≥ 1 kPa, aged: G*/sinδ ≥ 2.2 kPa) [61], and the temperature at which the sample just failed the criteria was recorded.

2. MSCR

MSCR tests were conducted to evaluate the rutting resistance and elastic recovery of asphalt binders at high temperature. MSCR, the most recent test introduced by the Federal Highway Administration, has demonstrated a stronger correlation with the rutting performance of hot mix asphalt when compared to the Superpave rutting parameter (G*/sinδ) [62,63]. MSCR measures the non-recoverable creep compliance (J_nr_) and the percentage of recovery (%R). J_nr_ is an indicator for rutting resistance, with the smaller J_nr_ showing the greater binder resistance against permanent deformation and rutting. The percentage of recovery shows the elasticity response of asphalt binders to recover after deformation. The test was performed following the guidelines of AASHTO T350 [64]. The test was conducted on the aged samples and the test temperature was 64 °C. The samples were subjected to testing at two stress levels (0.1 and 3.2 kPa) with a loading cycle of 1 s followed by a recovery cycle of 9 s. Twenty cycles of creep and recovery were applied at 0.1 kPa, and then ten cycles were applied at 3.2 kPa. The initial ten cycles at 0.1 kPa were used as pre-conditioning cycles [65]. The non-recoverable creep compliance (J_nr_) and the percentage of recovery (%R) at each stress level were reported. Each sample underwent two tests, and the report presents the average results.

#### 2.7.2. Frequency Sweep Test

To study the rheological behavior of asphalt binders at different temperatures and frequency ranges, a frequency sweep test was conducted using DSR. The results were used to generate the black diagram and Cole–Cole plots [66]. The test was carried out at temperatures of 6, 15, 25, 40, 60, and 80 °C, encompassing a broad spectrum of low to high temperature conditions. The frequency range was from 0.1 rad/s to 100 rad/s, comprising a total of 23 frequencies. The shear strain was kept at 0.01% to remain within the linear viscoelastic range.

## 3. Results and Discussion

### 3.1. Characterization of the Waste Plastic Bags

#### 3.1.1. Chemical Characterization Analysis Using FTIR

**Figure 2:**
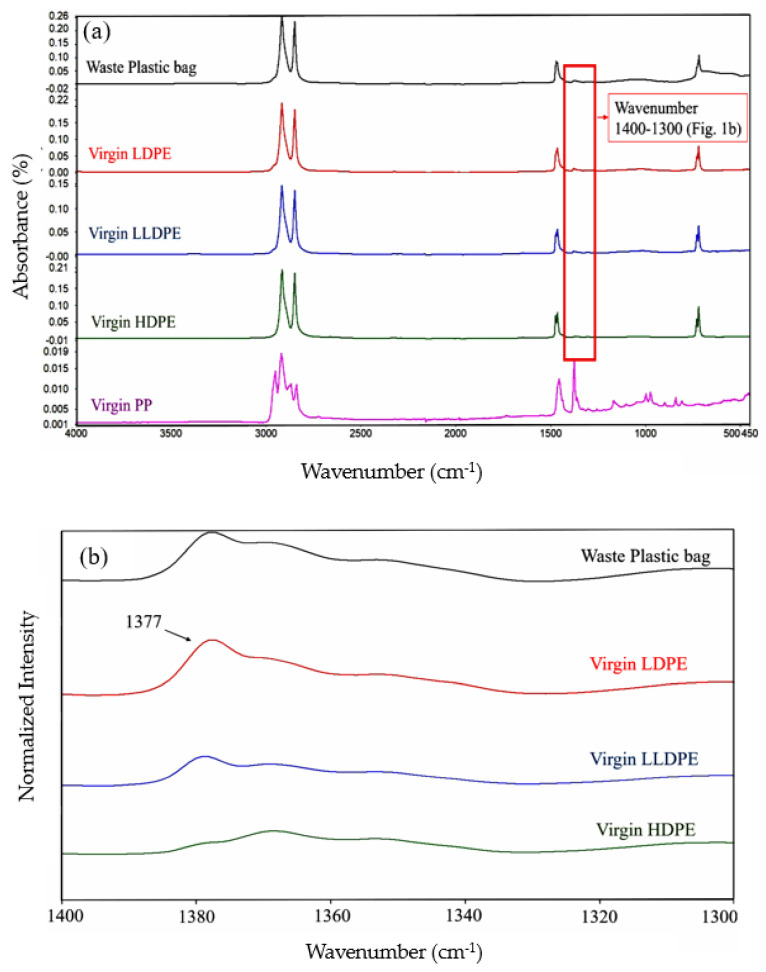
FTIR spectra of the waste plastic bags, virgin LDPE, LLDPE, HDPE, and PP at regions (**a**) 4000–450 cm^−1^, (**b**) 1400–1300 cm^−1^.

The FTIR spectra for the waste plastic bags, virgin LDPE, LLDPE, HDPE, and PP are shown in Figure 2a. The wavenumber and the vibrational mode of the chemical groups in virgin LDPE, LLDPE, and PP from the ATR-FTIR spectra are listed in Table 3. All identified peaks closely corresponded to the characteristic peaks of LDPE, LLDPE, HDPE, and PP reported in the literature.

Polypropylene and polyethylene spectrums can easily be distinguished. PP has methyl and methylene groups and has multiple peaks between 3000 and 2850 cm^−1^, whereas polyethylene contains methylene groups only and has just two C-H stretching peaks in this range [73,74]. This analysis confirms the absence of PP in the waste plastic sample.

As depicted in Figure 2a, the spectra of all polyethylene plastics, HDPE, LDPE, and LLDPE share significant similarities. Distinguishing between HDPE, LDPE, and LLDPE via FTIR is challenging due to their highly similar spectra. However, a subtle difference exists between HDPE and the other two plastics in a peak between 1400–1300 cm^−1^ (Figure 2b). Typically, HDPE lacks a peak at 1377 cm^−1^, while LDPE and LLDPE have a peak in this range [72]. This peak is attributed to the methyl group due to higher branching in LDPE and LLDPE than HDPE. In this study, the waste plastic sample exhibits a peak at this point (Figure 2b), indicating the presence of LDPE or LLDPE. The presence of HDPE cannot be confirmed using FTIR alone. Therefore, DSC was conducted on the samples as a complementary test to identify the composition of the waste plastic sample.

Additionally, there is no infrared signal at 1170 and 1167 cm^−1^, corresponding to the formation of C-O (carbonyl) from ester groups. Carbonyl formation is an indicator for the aging of polyethylene plastics. The absence of this peak indicates that the waste sample has undergone no or limited degradation during its service life [67].

#### 3.1.2. Thermal Behavior Analysis Using DSC

**Figure 3:**
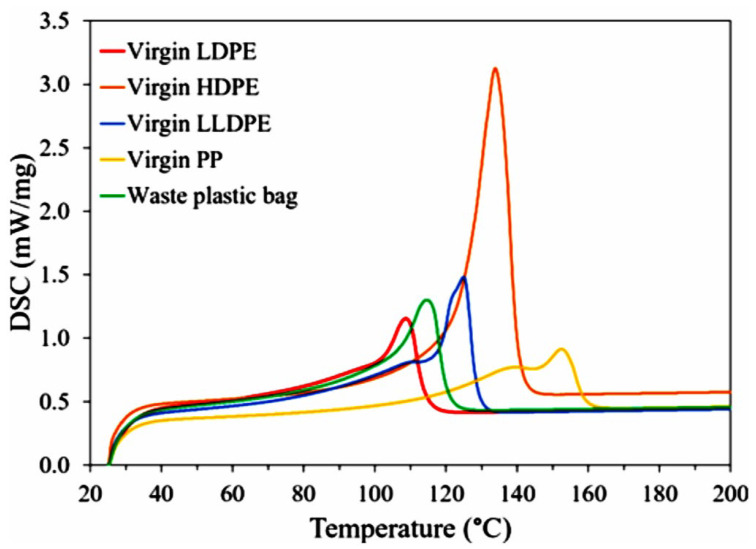
DSC analysis of virgin plastics and the waste plastic bags.

The DSC machine was employed as a complementary method to determine the plastic polymers present in the composition of the waste plastic bags. DSC proves highly effective in characterizing polyolefins via differentiation of the components based on their different thermal behavior [35]. Given the possibility of the presence of multiple polymers in the waste sample, DSC analyses were conducted on both the waste sample and the virgin polymers. The results from virgin plastics were then compared with those of the waste sample to characterize its composition. The DSC curves are presented in Figure 3.

The DSC curves of the virgin plastics showed a single peak which represents the melting point of each plastic. The values were at 110, 124, 130, and 160 °C for virgin LDPE, LLDPE, HDPE, and PP, respectively. Examination of the DSC curve for the waste plastic indicates the absence of peaks around 130 and 160 °C, confirming the absence of HDPE and PP in this sample. The DSC analysis of the waste sample revealed a single peak situated between the melting points of LDPE and LLDPE. This peak suggests that the waste plastic is a mixture of LDPE and LLDPE rather than consisting solely of one of them. The presence of a single peak, as opposed to two distinct peaks corresponding to each plastic’s melting point, indicates total miscibility of the two plastics in the waste sample [75].

According to the melting enthalpy (ΔH_m_) determined from the areas of the melting peaks in the DSC curves, the crystallinity of virgin LDPE, LLDPE, and the waste plastic samples was calculated. The degree of crystallinity was 18.8%, 29.2% and 30%, respectively. The slight increase of the crystallinity of the waste sample compared to virgin plastics could be due to the presence of additives or physical aging of the plastic bags during processing or service life.

#### 3.1.3. Thermal Stability Analysis Using TGA

**Figure 4:**
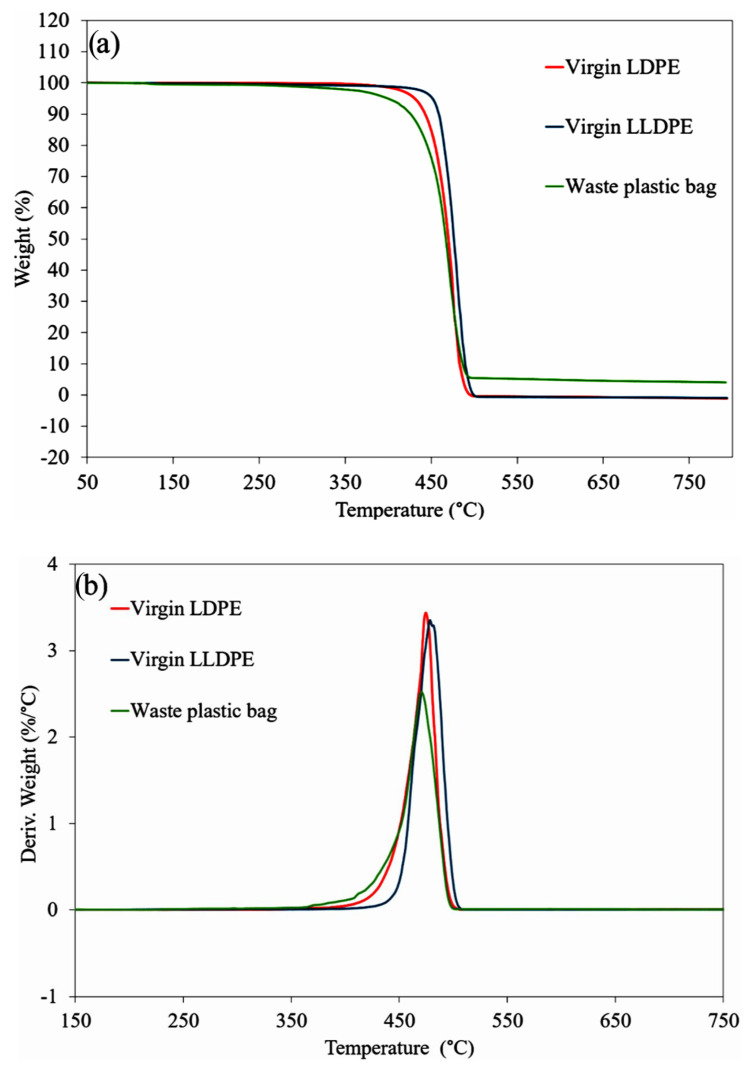
(**a**) TGA and (**b**) DTG analysis of the waste plastic bags and their virgin components.

To determine the presence of non-polymeric impurities in the waste sample, the thermal stability of the virgin LDPE and LLDPE, and the waste plastic was investigated using a TGA instrument. Figure 4a,b depict the TGA curves and the corresponding DTG curves of the samples. The TGA curves for virgin LDPE and LLDPE revealed a one-step degradation process, starting at around 400 °C, with complete volatilization occurring at temperatures below 500 °C. The TGA results highlight slightly different degradation behaviors between LDPE and LLDPE. LDPE undergoes degradation at lower temperatures compared to LLDPE, potentially attributed to the higher degree of branching in LDPE, providing a greater proportion of reactive tertiary carbons during the initiation step of degradation [76].

Degradation before 200 °C is due to thermal degradation of organic additives [77]. In the case of the waste plastic sample, a mass loss of 0.8% was observed at 200 °C, which was absent in the virgin LDPE and LLDPE samples. In this waste plastic sample, these organic additives can be attributed to the presence of black and green dyes coated on the plastic layer (Figure 4a). DTG curves show the T_max_ of the samples, which were 474.3 °C and 481.4 °C for LDPE and LLDPE, respectively, and for the waste plastic sample, it was 470.3 °C. The faster degradation rate of the waste plastic compared to the virgin plastics indicates a lower thermal stability for the waste plastic in comparison to its virgin counterparts.

Comparing the residues after TGA, it was observed that both virgin plastics exhibited complete degradation with zero residue. Conversely, the waste plastic had a 5.3% residue indicating the inclusion of inorganic additives and fillers which are commonly found in commercial plastic bags [78,79,80].

### 3.2. Size Distribution

The characterization analyses of the waste plastic revealed that the primary polymeric components of the sample are LLDPE and LDPE. For bitumen modification purposes, the virgin LDPE and LLDPE were used alongside the waste plastic sample to detect potential variations in bitumen performance that can be caused by the compositional differences within a waste plastic sample.

Waste plastic bags were shredded by the industry partner into particle sizes ranging from 0.3 to 0.6 cm for convenient dispersion in bitumen. Figure 1a displays an image of the shredded waste plastic bags, revealing particles with a mixture of white, black, and green pigments. Additionally, Figure 1b and c depict camera images captured from virgin LDPE and LLDPE pellets, respectively, with pellet sizes ranging from 0.2 to 0.25 cm.

### 3.3. Conventional Properties of Asphalt Binders

**Figure 5:**
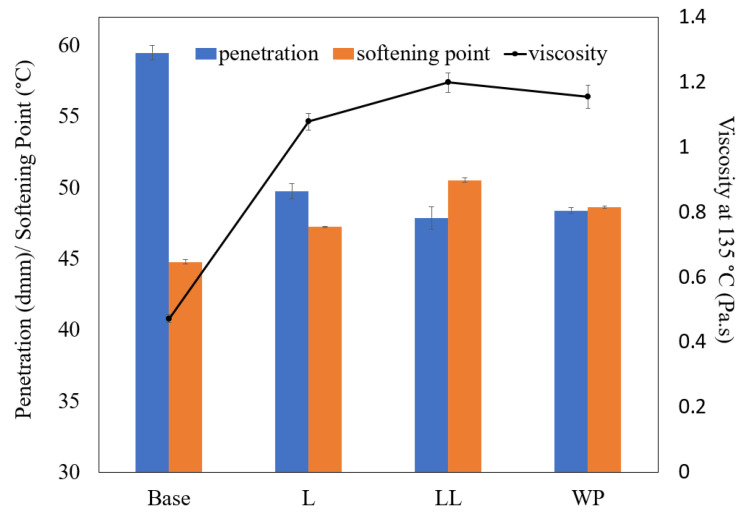
Conventional properties of asphalt binders.

Figure 5 displays the softening point, penetration, and viscosity of base bitumen and the modified binder samples. The base bitumen exhibited a softening point of around 44.8 °C. Incorporating virgin LLDPE for bitumen modification increased the softening point to 50.5 °C, signifying its enhanced resistance to rutting. Bitumen modified with virgin LDPE had a softening point of 47 °C. Comparing with the modification with LDPE, the softening point results imply that modification of bitumen with LLDPE is more effective in enhancing the performance of bitumen against permanent deformation. Considering the superior mechanical properties of LLDPE compared to LDPE [81], its incorporation into bitumen contributes to enhanced performance relative to LDPE. Where the waste plastic was used for bitumen modification, it resulted in a softening point of around 48.6 °C, indicating a slightly better performance compared to the virgin LDPE (+3.4%) and slightly lower performance than virgin LLDPE (−3.7%).

Regarding the penetration test results, the base bitumen had a penetration of 59.5 dmm. The introduction of all modifiers resulted in a reduction in penetration, indicating an increase in hardness. Modification with LLDPE led to a decrease in bitumen penetration to 47.9 dmm, and LDPE modification decreased it to approximately 49.8 dmm, showing the slightly higher ability of virgin LLDPE for improving the hardness of bitumen compared to virgin LDPE. Modification with the waste plastic sample resulted in a penetration value of 48.4 dmm, which was between the results that were obtained via LLDPE modification (+1.04% change) and virgin LDPE modification (−2.81% variation).

Concerning the viscosity of the binders, the base bitumen demonstrated a viscosity of 0.47 Pa·s. When modified with virgin LLDPE, the viscosity increased to 1.20 Pa·s, nearly 2.5 times that of unmodified bitumen. Bitumen modified with virgin LDPE exhibited a viscosity of 1.08 Pa·s, reflecting a less pronounced increase compared to LLDPE. When the waste plastic bags were employed as the bitumen modifier, the resulting modified bitumen showed a viscosity increase to 1.15 Pa·s, representing a 6.5% higher viscosity compared to virgin LDPE-modified bitumen and a 4% lower viscosity than virgin LLDPE-modified bitumen.

The results of the softening point, penetration, and viscosity were consistent in indicating variations observed via modification with the waste plastic compared to its virgin plastic counterparts. The waste plastic bags exhibited a performance level between that of bitumen modified with virgin LDPE and bitumen modified with virgin LLDPE.

### 3.4. Rutting Resistance Analysis

#### 3.4.1. Superpave Rutting Parameter

**Figure 6:**
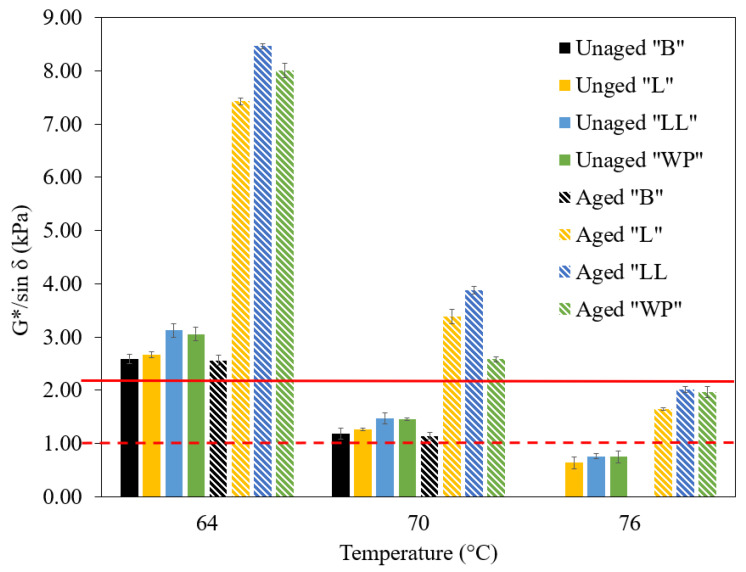
The rutting parameter (G*/sinδ) of bitumen and modified bitumen samples; the solid red line and dashed red line represent the maximum limit of the rutting parameter for unaged (2.2 kPa) and aged samples (1 kPa), respectively.

The Superpave rutting parameter (G*/sinδ) for both unaged and aged samples was assessed at temperatures of 64, 70, and 76 °C, as depicted in Figure 6. The figure illustrates a noticeable decrease in rutting resistance for all samples as temperatures rise. The base bitumen met the test criteria for both aged and unaged conditions at 64 °C but failed at 70 °C, establishing its high temperature performance grade as 64 °C based on AASHTO specification M320 [61]. Modification with LDPE, LLDPE, and waste plastic resulted in an obvious enhancement in the rutting parameter, with all modified binders meeting the criteria for passing the test at 70 °C in both aged and unaged conditions. Consequently, the high temperature performance grade of the bitumen was elevated to 70 °C, representing an improvement of one grade. This shows that the waste plastic was as effective as its virgin counterparts, LDPE and LLDPE, at enhancing the performance grade of the bitumen. Comparing the failure temperature of the rutting parameter tests (the temperature at which the rutting parameter of unaged binder is 2.2 kPa), it was observed that LLDPE-modified bitumen tolerated higher temperatures compared to LDPE modification The failure temperature was 73.5 °C for binder “LL”, 72 °C for binder “L”, and 73.4 °C for binder “WP”. It can be inferred that the incorporation of waste plastic led to a +2% variation in the rutting parameter compared to virgin LDPE modification, with a negligible difference observed compared to virgin LLDPE modification (−0.1%). The same sequence was observed for aged samples of the three binders. The improved stiffness of the bitumen modified with LLDPE and the waste sample compared to LDPE could be due to the higher crystalline level of LLDPE and waste plastic bag compared to LDPE. The crystalline area of polyethylene has high stiffness, acting as a reinforcing bar and consequently improves the rutting resistance of modified bitumen [13].

#### 3.4.2. MSCR

**Figure 7:**
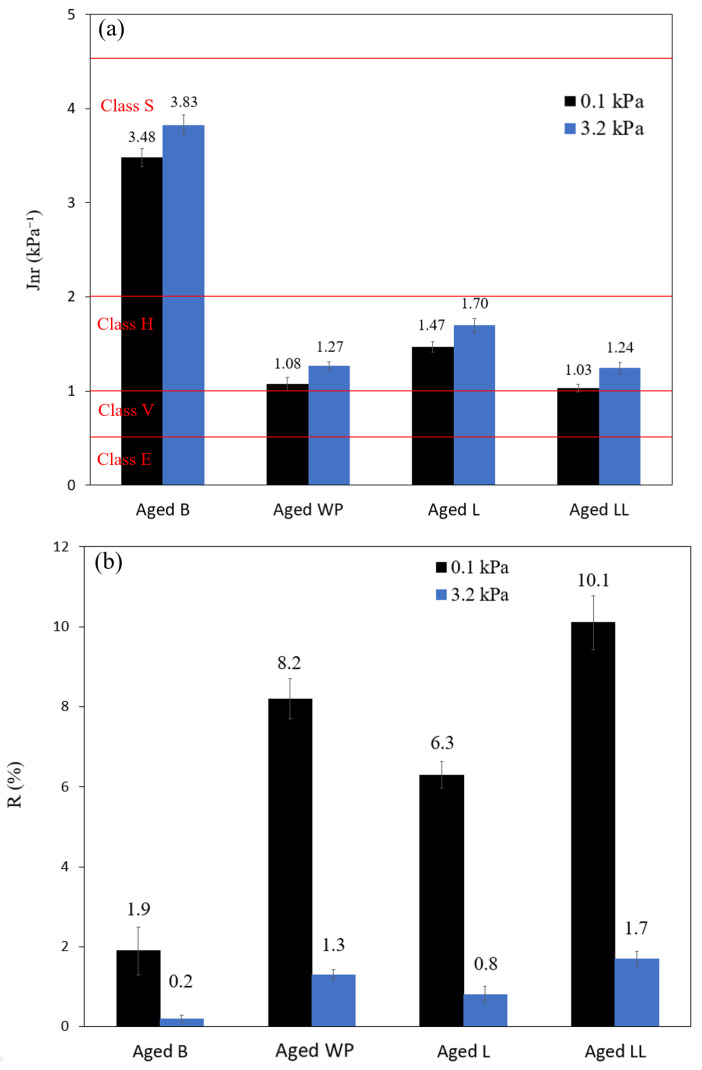
Non-recoverable creep compliances (J_nr_) (**a**) and percent recovery (R) (**b**) of aged bitumen and modified bitumen samples at 64 °C.

In addition to the Superpave rutting parameter (G*/sinδ), the MSCR test was conducted to evaluate the rutting resistance and elastic response of the asphalt binders. The MSCR, in comparison to the rutting parameter, effectively captures the rheological behavior of modified asphalt binders and highlights distinctions between the elastic response of different modifier types and their concentrations [62]. The results of the MSCR, J_nr_, and R% are presented in Figure 7a and 7b, respectively. The percentage of recovery serves as an indicator of the level of elasticity of asphalt binders, with higher values denoting greater elasticity. J_nr_ represents the unrecoverable creep compliance of the asphalt binder at high temperatures, where lower values indicate superior resistance to permanent deformation [82]. According to the AASHTO M332 specification [64], J_nr_ at 3.2 kPa is used to classify asphalt binders based on the suitability for four different traffic loadings. The category starts from standard traffic (S) (>70 km/h), high traffic (H) (20–70 km/h), very high traffic (V) (<20 km/h), and extreme traffic (E) (<20 km/h). The required J_nr_ for each classification is as follows: max 4.5 kPa^−1^, max 2 kPa^−1^, max 1 kPa^−1^, and max 0.5 kPa^−1^, respectively [83]. As shown in Figure 7a, the J_nr_ for base bitumen is 3.83 kPa^−1^, indicating that base bitumen can withstand rutting for the standard traffic level “S”. Modification with both virgin and waste plastics led to a decrease in J_nr_, indicating an enhancement in rutting resistance of the binder. Specifically, modification with virgin LLDPE and the waste plastic reduced the J_nr_ to 1.24 and 1.27 kPa^−1^, respectively. LDPE modification resulted in a reduction of J_nr_ to 1.7 kPa^−1^. All modifications demonstrated an improvement in the allowable traffic loading of the binder, transitioning from standard traffic “S” to heavy traffic level “H”.

Regarding the elastic recovery of the binders (Figure 7b), the values of the percentage of recovery for all binders decreased with increasing the creep stress from 0.1 to 3.2 kPa, indicating that binders became more viscous under heavy traffic load [82]. Comparing the age of the recovery values at 3.2 kPa, the base bitumen exhibited negligible recovery (0.2%). Modification of the bitumen with both virgin and waste plastic led to a small improvement of elasticity. Modification with virgin LLDPE and waste plastic demonstrated similar recovery results, 1.7 and 1.3%, respectively. LDPE modification resulted in a limited improvement of elasticity response with an elastic recovery of 0.8%. Comparing the results shows that the variation in the percentage of recovery between modification with the waste sample and its virgin counterparts, LLDPE and LDPE, was −23% and +62%, respectively.

### 3.5. Rheological Analysis Using Frequency Sweep Test

#### 3.5.1. Black Diagram

A black diagram is a graphical representation of the complex modulus, G*, plotted against the phase angle, δ. With elimination of the influence of frequency and temperature, the plot provides a comprehensive presentation of all data in a singular graph [84].

**Figure 8:**
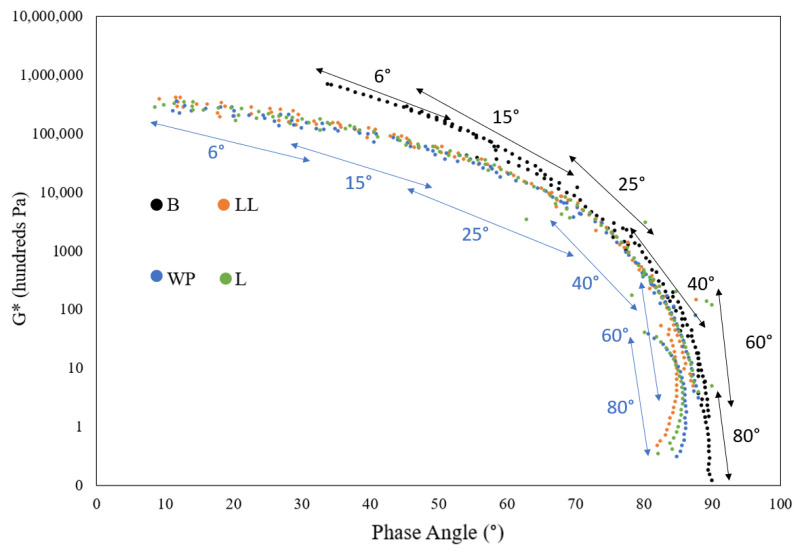
Black diagrams of base bitumen and modified binders.

Figure 8 shows the black diagrams of base bitumen and the modified binders. Each curve on the black diagram in Figure 8 corresponds to one of the six temperatures used in the test (5, 15, 25, 40, 60, and 80 °C). As shown by blue arrows in Figure 8, the lower temperatures are positioned on the left side of the black diagram, with the results at higher temperatures progressively displayed towards the right. Increasing the temperature led to an elevation in the phase angle of the binders, indicating a transition towards a softer, more viscous material [85]. The base bitumen had the highest phase angle for all the temperature ranges. Samples modified with virgin LLDPE, LDPE, and WP demonstrated similar behavior across all tested temperatures and frequency ranges. As depicted in the black diagram, the phase angles of the base binder at temperatures of 60 °C and 80 °C are 85–90 and 90, respectively, indicating a transition to a purely viscous material [86]. This transition occurred at a slower rate for the modified binders, as evidenced by the phase angle being shifted to the left side of the graph for these binders, positioning the black diagram in a less viscous domain. All modified binders exhibited similar phase angle values at temperatures of 60 (δ = 80–85) and 80 °C (δ = 80–85). Additionally, at these temperatures, the complex modulus of the modified binders was greater than those of the base bitumen. At 60 and 80 °C, the base bitumen had a complex modulus ranging between 150–19,000 and 12–1700 Pa, respectively. With modifications, the complex modulus nearly doubled, with LLDPE showing slightly higher values compared to WP and LDPE. An increased complex modulus and decreased phase angle at high temperatures is an indicator of the improved elasticity and stiffness of the binder, respectively, which shows a greater rutting performance of the binder after modification [87].

#### 3.5.2. Cole–Cole Diagram

**Figure 9:**
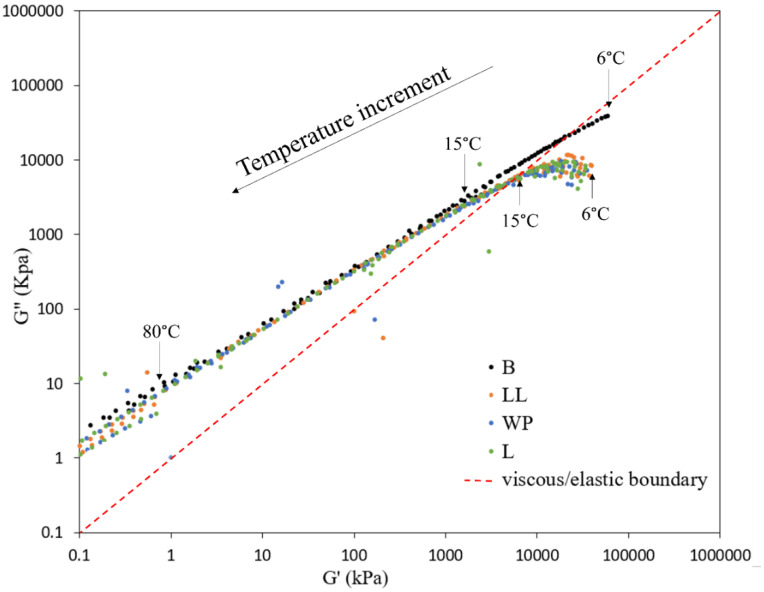
Cole–Cole diagram of bitumen and modified bitumen samples.

Figure 9 shows Cole–Cole plots with G” as a function of G’ for base bitumen and bitumen modified with virgin and waste plastics. Cole–Cole diagrams are utilized to illustrate the relationship between the storage modulus (G’) and loss modulus (G”) concerning the frequency for the applied test load [88]. The relationship helps to identify the composition of the material elasticity (storage modulus, G’) and viscosity (loss modulus, G”). In Cole–Cole plots, the 45° line signifies the point at which G’ and G” are equal, indicating that the binder exhibits viscoelastic properties (as shown by the dotted line in Figure 9). Any material positioned below this line indicates an elastic-dominant phase (solid behavior), whereas any material located above this line suggests a predominantly viscous phase (liquid behavior) [89].

As shown in Figure 9, there is a linear relationship between the storage modulus and loss modulus, and both decrease with temperature increment. All the data points of base bitumen, except for a small part at 6 °C, are concentrated above the 45° line, indicating the dominant viscous behavior of base bitumen. By adding virgin LDPE, LLDPE, or waste plastic, the curve moved closer to the boundary between the viscous and elastic domain, indicating the improvement of the elasticity of modified bitumen. Furthermore, the curves for binders modified using virgin LLDPE, virgin LDPE, and the waste plastic are very similar, which indicates that the waste plastic performs as effectively as the virgin samples in enhancing the elastic properties of bitumen.

### 3.6. Effect of Short-Term Aging on Rheological Properties

Asphalt binders experience an increase in stiffness and a reduction in their viscous properties as they undergo aging [90]. Greater susceptibility of the binder to aging results in increased brittleness of asphalt mixtures over their service life [91]. To study the effect of modifications on resistance of the binders against aging, the crossover modulus of the samples was studied using black diagrams of the aged and unaged samples. The crossover modulus is the modulus at which the phase angle (δ) equals 45°, expressing that the storage modulus (G’) and loss modulus (G”) have equal magnitudes. A binder with δ < 45° indicates more solid behavior, while δ > 45° suggests a more fluid behavior in the material [92]. The crossover modulus is a valuable rheological parameter for quantifying susceptibility to aging [93]. Previous studies have consistently demonstrated that as bitumen undergoes aging, its stiffness tends to increase, resulting in a decrease in the crossover modulus [94,95]. Binders with higher susceptibility to aging exhibit more significant variations in the crossover modulus before and after the aging process.

**Figure 10:**
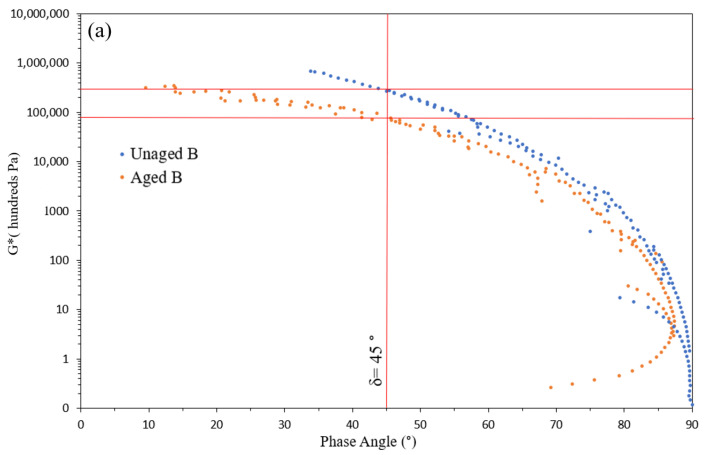

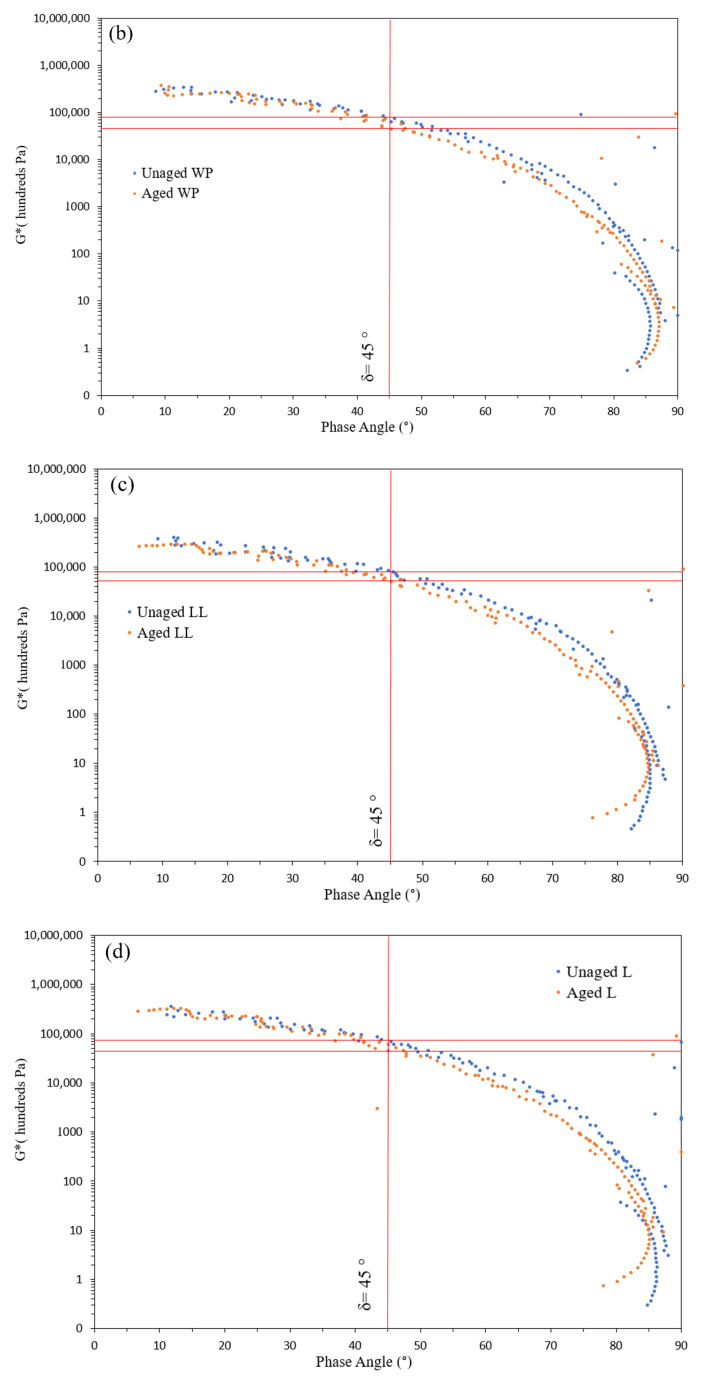
Crossover modulus changes before and after aging for base bitumen (**a**), binder WP (**b**), binder LL (**c**), and binder L (**d**).

Figure 10 illustrates the black diagrams of both unaged and aged samples for all binders. Through the aging process, a distinct shift of each binder’s black diagram towards lower phase angles was observed. These shifts demonstrate a change towards a stiffer and less viscous material. By comparing the curves, it becomes evident that unmodified bitumen displays the highest variance in the crossover modulus. The substantial reduction in the crossover modulus for base bitumen indicates its higher susceptibility to aging, suggesting its greater potential for distresses and failures such as fatigue and thermal cracking [93,95].

The curves of the aged samples for modified binders exhibit limited variation in the crossover modulus compared to the base bitumen. This observation highlights improvement in the age-resistant properties of bitumen after modification. The values for the crossover modulus before and after aging are shown in Table 4. The reduction in the crossover modulus after aging for the base bitumen was −70.4%. Comparing the modified binders, LL exhibited the smallest reduction in the crossover modulus (−35%), followed by binder L (−40%). Binder WP showed a slightly higher variation in the crossover modulus compared to the other two (−43.7%). These results suggest a slightly higher effectiveness of the virgin plastic, as opposed to the waste sample, in enhancing the binder’s resistance against aging, which can be attributed to impurities introduced by additives present in the waste sample.

### 3.7. Thermal Stability

**Figure 11:**
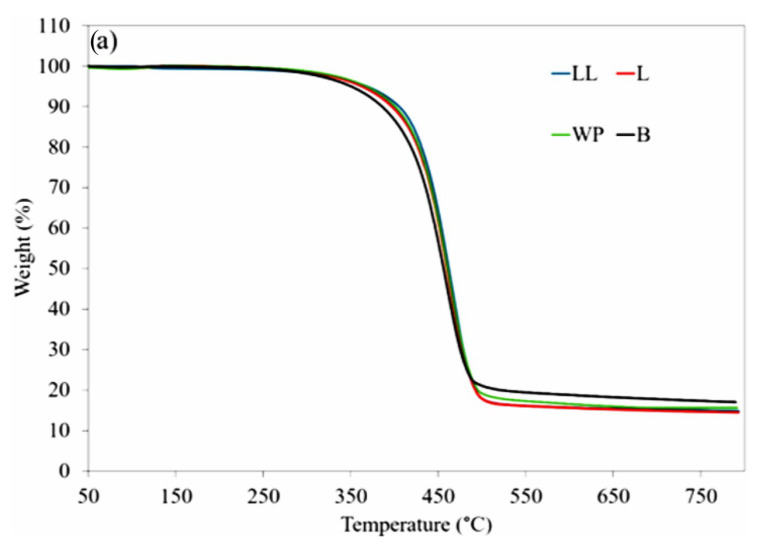

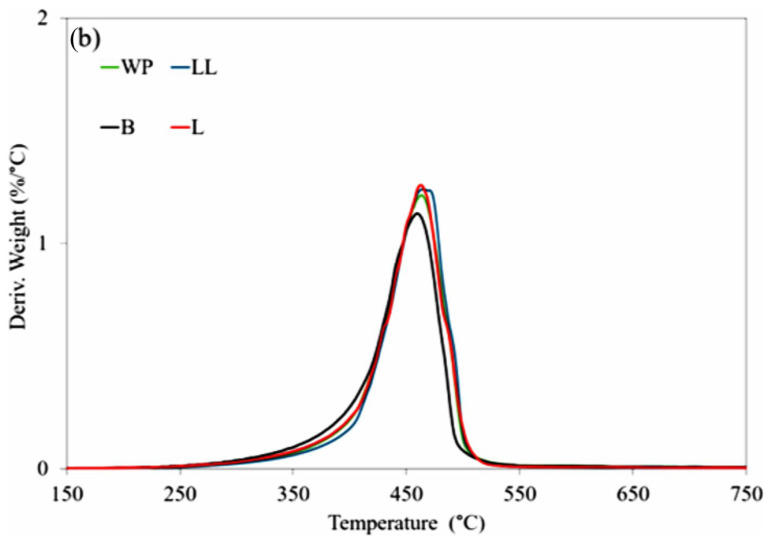
(**a**) TGA and (**b**) DTG analysis of neat bitumen and modified bitumen samples.

The TGA curves of base bitumen and the modified binders are also depicted in Figure 11a and 11b, respectively. The corresponding values for the maximum decomposition temperature (T_max_) and the residue at 750 °C are listed in Table 5. In contrast to plastics, which undergo a single-phase thermal decomposition (Figure 4), bitumen undergoes a two-phase thermal decomposition process. The initial phase, occurring between 250 to 400 °C, leads to the loss of lighter fractions, saturates, and aromatics. The second phase, spanning from 450 to 750 °C, involves more intricate reactions, degrading heavier fractions, resins, and asphaltenes [96]. The temperature corresponding to the maximum weight loss rate in the second phase was 460 °C. Beyond 750 °C, the TGA curve becomes linear, indicating negligible thermal degradation. The residual content of bitumen at 750 °C is about 17%, and according to literature, this remaining mass loss of bitumen is associated with asphaltenes [97].

The TGA graph shows that the base bitumen is degrading faster than the modified binders, showing the modifications have improved the thermal stability of the bitumen. This is due to the superior thermal stability of the plastics over bitumen, the addition of which to bitumen has improved its stability. Among the modified binders, LL had higher thermal stability than L and WP which showed comparable thermal degradation. Regarding the residue at 750 °C, the final residue is higher for the WP sample (15.8%) compared to the L (14.9%) or LLDPE sample (14.6%). As previously discussed regarding the thermal degradation of plastics, this difference is attributed to the presence of inorganic additives in the WP sample, influencing the thermal behavior of the resulting modified bitumen.

While the extreme temperatures employed in TGA testing may not precisely replicate real-world conditions, the data derived from these tests serve a crucial role in forecasting the long-term performance and thermal resistance behavior of both the base bitumen and the modified bitumen samples in field conditions.

## 4. Conclusions

The use of waste plastics in modifying asphalt binders has gained considerable attention. Plastic products are commonly composed of a mixture of different plastics, a practice adopted to enhance specific asphalt properties. Each component has a distinct impact on the properties and performance of the bitumen, resulting in variations in the performance of the modified binder.

In this investigation, unidentified waste of plastic bags was selected for modification of bitumen. The study explored the feasibility of utilizing the waste plastic for bitumen modification as an alternative to virgin plastics, with a focus on maintaining the performance integrity of the binder. The detailed characterization of the waste sample unveiled its composition as a mix of two plastics: LDPE and LLDPE. Further analysis of thermal stability confirmed that nearly 93.9% of the composition consisted of plastic polymers, with 0.8% attributed to organic additives and 5.3% to inorganic additives.

The study highlighted the potential of waste plastic bags as an effective bitumen modifier to enhance the rutting, aging, and thermal resistance of the resultant modified bitumen. The findings indicated that the waste plastic’s multicomponent nature, comprising of a mix of LDPE and LLDPE, did not cause any changes in the grading of the modified binder when compared to virgin LDPE and LLDPE. As per the Superpave rutting parameter outcomes, modifications with both the waste plastic and its virgin counterparts, LDPE and LLDPE, increased the high temperature performance grade of the modified bitumen by one grade, shifting from 64 to 70 °C. Based on the MSCR results, all modifications demonstrated an enhancement in the allowable traffic conditions of the binder, progressing from “standard” to “heavy” traffic.

The softening point, viscosity, and penetration values of the bitumen modified with the waste plastic bags were positioned between the outcomes observed for modifications using virgin LDPE and LLDPE. The variance between waste and virgin plastics was more pronounced using MSCR. In these aspects, the waste plastic bags behaved very similarly to LLDPE rather than LDPE in enhancing the rutting resistance and elastic properties of the modified binder.

All three modified binders had higher thermal and aging stability than the base bitumen. However, the bitumen modified using the waste plastic exhibited lower thermal stability compared to bitumen modified using virgin LDPE and LLDPE. This was attributed to the organic and inorganic impurities of the waste plastic.

This research demonstrated that the analyzed plastic bag waste is suitable for use in binder modification, serving as a viable alternative to virgin LLDPE. The knowledge obtained from this research could be further explored to investigate the emissions and the release of micropollutants arising from the use of asphalt mixtures containing waste plastics.

## Figures and Tables

**Table 1:** Summary of plastic polymers properties.

Plastic Type	Grade	Density at 25° C (g/cm^3^)	Melting Point (°C)
PP	SM598	0.9	160
LDPE	LD2426K	0.924	110
HDPE	HD1010J	0.956	127
LLDPE	LL7410D	0.921	121

**Table 2:** Compositions of the asphalt binders.

Binder Name	Modifier Content per Weight of Bitumen (%)	Modifier Composition (wt.%)		
		Virgin LLDPE	Virgin LDPE	Waste Plastic Bag
B	0	0	0	0
LL	5	100	0	0
L	5	0	100	0
WP	5	0	0	100

**Table 3:** ATR-FTIR characteristic of the virgin plastics.

Plastic Type	Wavenumber (cm^−1^)	Chemical Group and Vibrational Mode	Reference
PP	2950	asymmetrical stretching vibrations of CH_3_	[67,68]
	2917	asymmetrical stretching vibrations of CH_2_	[67,68]
	2869	stretching vibrations of CH_3_	[67,68]
	1456	symmetrical bending vibrations of CH_3_	[67,68]
	1376	symmetrical bending vibrations of CH_3_	[67,68]
	1166	wagging vibrations of CH	[67,68]
		rocking vibrations of CH_3_	[67,68]
		stretching vibrations of C-C	[67,68]
	996	rocking vibrations of CH_3_	[67,68]
	973	rocking vibrations of CH_3_	[67,68]
		stretching vibrations of C-C	[67,68]
	840	rocking vibrations of CH	[67,68]
	808	stretching vibrations of C-C	[67,68]
LDPE/LLDPE	2917	asymmetric stretching vibrations of CH_2_	[69,70,71]
	2848	symmetric stretching vibrations of CH_2_	[69,70,71]
	1471	bending deformation vibration of CH_2_ (crystalline domains)	[69,70,71]
	1463	bending deformation vibration of CH_2_ (amorphous domains)	[69,70,71]
	1377	symmetric deformation vibration of CH_3_	[69,70,71]
	1369	wagging deformation vibration of CH_2_	[69,70,71]
	729	rocking deformation vibrations of CH_2_ in crystalline domains	[69,70,71]
	718	rocking deformation vibrations of CH_2_ in amorphous domains	[69,70,71]
HDPE	2917	asymmetric stretching vibrations of CH_2_	[67,72]
	2848	symmetric stretching vibrations of CH_2_	[67,72]
	1471	bending deformation vibration of CH_2_ in crystalline domains	[67,72]
	1463	bending deformation vibration of CH_2_ in amorphous domains	[67,72]
	729	rocking deformation vibrations of CH_2_ in crystalline domains	[67,72]
	718	rocking deformation vibrations of CH_2_ in amorphous domains	[67,72]

**Table 4:** Crossover modulus of binder before and after aging.

Binder Name	Crossover Modulus (MPa)		Crossover Changes after Aging (%)
	before Aging	after Aging	
B	27	8	−70.4
WPE	8	4.5	−43.7
L	7.5	4.5	−40
LL	8	5.2	−35

**Table 5:** TGA and DTG thermogram data of asphalt binders.

Binder Name	T_max_	Residue at 750 °C (%)
B	459.3	16.9
L	462.2	14.9
LL	464.9	14.6
WP	462.8	15.8

## Data Availability

Data are contained within the article.
